# Neuronal hibernation following hippocampal demyelination

**DOI:** 10.1186/s40478-021-01130-9

**Published:** 2021-03-01

**Authors:** Selva Baltan, Safdar S. Jawaid, Anthony M. Chomyk, Grahame J. Kidd, Jacqueline Chen, Harsha D. Battapady, Ricky Chan, Ranjan Dutta, Bruce D. Trapp

**Affiliations:** 1 Department of Neurosciences, Lerner Research Institute, Cleveland Clinic, 9500 Euclid Avenue/NC30, Cleveland, OH 44195 USA; 2 Present Address: Department of Perioperative Medicine, Oregon Health and Science University, Portland, OR 97239 USA; 3 Present Address: Department of Pediatrics, School of Medicine, Case Western Reserve University, Cleveland, OH 44106 USA; 4 Present Address: Department of Biomedical Engineering, School of Medicine, Case Western Reserve University, Cleveland, OH 44106 USA; 5 Present Address: Imaging Institute, Cleveland Clinic, Cleveland, OH 44195 USA; 6 Cleveland Institute for Computational Biology, Cleveland, OH 44106 USA

**Keywords:** Hippocampal demyelination, Long-term potentiation (LTP), Dendritic spines, Transcript profiling

## Abstract

Cognitive dysfunction occurs in greater than 50% of individuals with multiple sclerosis (MS). Hippocampal demyelination is a prominent feature of postmortem MS brains and hippocampal atrophy correlates with cognitive decline in MS patients. Cellular and molecular mechanisms responsible for neuronal dysfunction in demyelinated hippocampi are not fully understood. Here we investigate a mouse model of hippocampal demyelination where twelve weeks of treatment with the oligodendrocyte toxin, cuprizone, demyelinates over 90% of the hippocampus and causes decreased memory/learning. Long-term potentiation (LTP) of hippocampal CA1 pyramidal neurons is considered to be a major cellular readout of learning and memory in the mammalian brain. In acute slices, we establish that hippocampal demyelination abolishes LTP and excitatory post-synaptic potentials of CA1 neurons, while pre-synaptic function of Schaeffer collateral fibers is preserved. Demyelination also reduced Ca^2+^-mediated firing of hippocampal neurons in vivo. Using three-dimensional electron microscopy, we investigated the number, shape (mushroom, stubby, thin), and post-synaptic densities (PSDs) of dendritic spines that facilitate LTP. Hippocampal demyelination did not alter the number of dendritic spines. Surprisingly, dendritic spines appeared to be more mature in demyelinated hippocampi, with a significant increase in mushroom-shaped spines, more perforated PSDs, and more astrocyte participation in the tripartite synapse. RNA sequencing experiments identified 400 altered transcripts in demyelinated hippocampi. Gene transcripts that regulate myelination, synaptic signaling, astrocyte function, and innate immunity were altered in demyelinated hippocampi. Hippocampal remyelination rescued synaptic transmission, LTP, and the majority of gene transcript changes. We establish that CA1 neurons projecting demyelinated axons silence their dendritic spines and hibernate in a state that may protect the demyelinated axon and facilitates functional recovery following remyelination.

## Introduction

The cellular complexity of the central nervous system (CNS) presents formidable challenges for investigating disease mechanisms. This is especially the case for multiple sclerosis (MS), which is a CNS disease characterized by demyelination of both white matter and gray matter and subsequent neurodegeneration [1–4]. The characteristics of white- and gray-matter lesions are distinct and impact neuronal function in different ways. Axons are transected during immune-mediated white-matter demyelination [5] and many chronically-demyelinated axons degenerate [6]. While neuronal degeneration and synaptic loss have been described in cortical and deep gray-matter lesions obtained from end-stage MS patients [3, 7–10], little is known about early changes in neuronal and synaptic function following gray-matter demyelination. A similar scenario exists for the responses of microglia and astrocytes, which are well-described in acute and chronic white-matter lesions [11–13] as well as chronic gray-matter lesions [3, 14], but are understudied in acute gray-matter lesions.

Cognitive dysfunction occurs in greater than 50% of individuals with MS, has a major impact on the quality of life of MS patients, and is a better predictor of occupational and social impairment than physical disability [15–19]. Reduced cognitive processing speed and episodic memory are the most frequently reported cognitive alterations in individuals with MS [20]. Hippocampal demyelination is a prominent feature of postmortem MS brains [9, 21] and hippocampal atrophy correlates with cognitive decline in MS patients [21–27]. While no single animal model recapitulates all aspects of MS, the rodent cuprizone model provides a reliable platform to investigate hippocampal demyelination and remyelination. When added to normal diet for 12 weeks, the oligodendrocyte toxin cuprizone demyelinates ~ 90% of the hippocampus and decreases memory/learning [9]. Upon removal of cuprizone from the diet for six weeks, ~ 60% of hippocampal myelin is replaced by remyelination and memory/learning is restored [9]. This supports the hypothesis that neuronal and synaptic function are altered by demyelination and restored by remyelination.

Long-term potentiation (LTP) of hippocampal CA1 pyramidal neurons is considered to be a major cellular mechanism for increased learning and memory in the mammalian brain [28]. In the CA1 region of the hippocampus, LTP induction is associated with a rise in postsynaptic Ca^2+^ caused by activation and modulation of glutamate receptors [29]. LTP modulators include altered release of pre-synaptic glutamate [30], alterations in post-synaptic sensitivity to glutamate [29], and altered astrocyte participation in the tripartite synapse [31]. In hippocampal slices obtained from mice demyelinated by cuprizone for 1–6 weeks, synaptic transmission in CA1 pyramidal neurons was diminished and CA1 neuronal firing rates were substantially reduced in vivo [32]. It remains to be determined whether hippocampal demyelination alters LTP.

This report compares changes in synaptic electrophysiology, dendritic spine ultrastructure, and gene transcripts between myelinated, demyelinated, and remyelinated hippocampi. We show that CA1 neurons in demyelinated hippocampi maintain dendritic spines that are ultrastructurally mature, but functionally silent. Gene transcripts altered by demyelination encode proteins that regulate myelination, innate immunity, synaptic signaling, and astrocyte participation in the tripartite synapse. We consider dendritic silencing to be a neuroprotective response that transiently mitigates degeneration of demyelinated axons. By maintaining the structural integrity of their synaptic spines, demyelinated CA1 neurons may facilitate synaptic function upon remyelination.

## Material and methods

### Cuprizone demyelination

All animal experiments were approved by the Institutional Animal Care and Use Committee (IACUC) of the Cleveland Clinic. Six-week-old C57/BL/6J male mice were purchased from Jackson Laboratory (Bar Harbor, Maine) and used for all experiments. Hippocampal demyelination/remyelination was induced as described previously [9, 33]. Mice were fed custom-made chow pellets (Harlan Teklad, Madison, WI) containing 0.3% cuprizone (bis-cyclohexanone oxaldihydrazone, Sigma-Aldrich, St. Louis, MO) for 12 weeks ad libitum. During this demyelination period, mice were given daily intraperitoneal injections of rapamycin (10 mg/kg body weight), which prevents spontaneous remyelination [33, 34]. Control mice were injected daily with rapamycin for 12 weeks. Following cuprizone treatment, mice were returned to normal chow (without rapamycin injections) to allow spontaneous remyelination for six weeks. Physiological studies included hippocampal slices obtained from mice sacrificed after demyelination (12 weeks of cuprizone treatment), remyelination (12 weeks of cuprizone followed by 6 weeks of normal diet), and appropriate aged-matched controls. Using three-dimensional electron microscopy (3D EM), we compared the ultrastructure of CA1 dendritic spines in myelinated, demyelinated (12 weeks of cuprizone treatment), and remyelinated (12 weeks of cuprizone followed by 6 weeks of normal diet) hippocampi. RNA-seq studies were performed at the same time points.

### Hippocampal slice electrophysiology

Experiments were performed in the CA1 region of 400 µm-thick transverse hippocampal slices as previously reported [32, 35–37]. Mice were decapitated after CO_2_ narcosis, and the brains were immediately removed and placed in saline kept near 0 °C. Hippocampi were quickly dissected and sliced with a McIlwain tissue chopper. Slices were stabilized in carbogenated (95% O_2_, 5% CO_2_) artificial cerebrospinal fluid (ACSF, in mM: 126 NaCl, 3.5 KCl, 1.3 MgCl_2_, 2 CaCl_2_, 1.3 NaH_2_PO_4_, 25 NaHCO_3_ and 10 glucose at pH 7.4) for 1–2 h at room temperature.

Experiments were performed in a Haas-type slice chamber where individual slices were kept at the interface of warm oxygenated ASCF (at 33–34 °C) continually flowing at a rate of 3–3.5 ml/min. For simultaneous extracellular recordings of excitatory post-synaptic potentials (EPSPs) and afferent volleys (AVs), glass microelectrodes filled with 2 M NaCl (resistance 1–2 MΩ) were placed in the CA1 stratum radiatum. Responses were evoked by stimulation of Schaffer collateral fibers by a bipolar tungsten wire electrode, with ~ 50-µsecond pulses at 30-s intervals. Evoked responses were recorded in the same layer by placing the bipolar recording electrode at a distance adjusted to yield clear AVs (presynaptic response) and consequent EPSPs (postsynaptic response). Tetanic LTP was elicited by double 1-s bursts of 100-Hz high-frequency stimulation (HFS) delivered 20 s apart. To obtain maximal LTP, HFS was repeated at least three times at 15-min intervals. To further ensure that impaired synaptic plasticity followed by loss of synaptic transmission were mainly due to the effects of demyelination on the post-synaptic terminal, presynaptic terminal function was further assessed by employing a paired-pulse facilitation (PPF) paradigm (Additional File 1: Fig. S1) over a range of interval durations between the evoked EPSPs (10, 50, 100, 200, and 400 ms). To obtain more comprehensive data, responses were recorded at a range of stimulus intensities before and after applications of HFS. Results from slices in the same experimental group were pooled and normalized to the maximal values of stimulus intensity and the corresponding amplitude of the AV or EPSP slope to plot synaptic input–output or time course data.

### In vivo magnetic resonance imaging (MRI)

MRI comparisons of myelinated (n = 10), demyelinated (n = 10), and remyelinated (n = 10) hippocampi were performed on a 9.4 T horizontal bore magnet (Bruker BioSpin, Bruker Corporation, Billerica, MA) using a 35 mm inner-bore diameter mouse radiofrequency coil. Hippocampal volume was quantified from T2-weighted (T2w) MRI, and hippocampal neuronal activity was quantified from manganese-enhanced MRI (MEMRI) [38, 39]. During scanning, mice were anesthetized using 1.5% isoflurane in O_2_ and body temperature and respiratory frequency were monitored and kept constant at 35 ± 1.5 °C and 60 ± 20 respiratory cycles/min, respectively. Imaging included a structural 3-dimensional (3D) T2w MRI and 3D T1-weighted MRIs acquired before (PreMn) and 24 h after (PostMn) 2 daily intraperitoneal injections of manganese chloride (50 mM MnCl_2_). Voxel size was identical for all scans: ~ 0.140 × 0.140 × 0.140 mm^3^.

All T2w images were corrected for intensity non-uniformities and extra-cerebral non-brain tissues were removed. Hippocampi were segmented from these T2w MRIs to quantify hippocampal volume using an in-house multi-atlas, registration-based segmentation pipeline. All modalities were registered to the T2w MRIs to obtain hippocampal metrics [40, 41]. The volume of MnCl_2_-enhanced voxels in the hippocampi (eVol_hippo_) was quantified by comparing the intensity of each individual PostMn voxel to the mean intensity of its local neighborhood of 6 voxels on the PreMn MRI. The voxels that survived false discovery rate correction [42] were included in the eVol_hippo_. MRI processing and quantification were performed using FSL and AFNI toolkits ([43]; https://afni.nimh.nih.gov).

### Tripartite synapse and dendritic spine ultrastructure

Mice were perfused with 4% paraformaldehyde and 2.5% glutaraldehyde in 0.1 M sodium cacodylate buffer. Brains were removed and the CA1 region of the hippocampus was isolated, stained with OsO_4_-ferricyanide, thiocarbohydrazide, aqueous OsO_4_, aqueous uranyl acetate, and Walton’s lead aspartate, then dehydrated and embedded in Epon (all reagents were obtained from Electron Microscopy Sciences, Hatfield, PA). Tissue blocks were imaged utilizing a Carl Zeiss Sigma VP scanning electron microscope (EM) containing a 3View in-chamber ultramicrotome system and a Gatan high sensitivity, low-kV backscattered electron detector (Gatan, Warrenvale, PA). Three to five hundred serial sections of primary dendrites located 50–100 µm from the neuronal cell body were collected at 5–7 nm per pixel resolution, 48 μm × 48 μm size and at 75 nm thickness. Videos of serial sections and spine 3D reconstruction can be found in the Supplemental material (Additional File 2 and 3: Videos S1–S2). Images were processed and registered using ImageJ software with FIJI plug-in sets. Image stacks were imported into Reconstruct software [44] and tracing of objects was used to obtain 3D reconstructions and meshes for analysis. The density, shape, and volume of dendritic spines, the area and shape of postsynaptic density (PSD), and the percent area of synaptic clefts opposed by astrocyte processes were quantified for at least 10 μm of each dendrite as described previously [45]. Fifteen dendrites from 3 mice were analyzed in myelinated hippocampi (716 spines) and twelve dendrites from 3 mice were analyzed in demyelinated (593 spines) and remyelinated (662 spines) hippocampi. Spine type (thin, mushroom and stubby) and PSD type (macular and perforated) analyses include nine dendrites from 3 myelinated (158 spines), 3 demyelinated (157 spines) and 3 remyelinated (178 spines) hippocampi as described previously [45].

### RNA sequencing

RNA was isolated from myelinated (n = 5), demyelinated (n = 5), and remyelinated (n = 5) mouse hippocampi using Qiagen RNAeasy kits following the manufacturer’s protocol (Qiagen Inc., Hilden, Germany). RNA-seq libraries were prepared with Illumina’s TruSeq Stranded Total RNA with Ribo-Zero Globin kit and sequenced on a HiSeq-2500 sequencer using Rapid Run v2, 100 bp, Paired-end run. Post-sequencing, raw demultiplexed fastq paired end read files were trimmed of adapters and filtered using the program skewer to throw out any with an average phred quality score of less than 30 or a length of less than 36. Trimmed reads were then aligned using the HISAT2 aligner to the *Mus musculus* NCBI reference genome assembly (v GRCm10). Aligned reads were counted and assigned to gene meta-features using the program feature Counts (Subread package). These count files were imported into the R software environment and were assessed for quality control, normalized, and analyzed using an in-house pipeline utilizing the edgeR Bioconductor [46] library for differential gene expression testing. Results were analyzed for differential expression using cufflinks, an RNA-Seq analysis package which reports the fragments per kilobase of exon per million fragments mapped (FPKM) for each gene. Differential genes were identified using a significance cutoff of FDR < 0.05. FKPM values for significantly-altered genes were used to generate heat maps in Morpheus matrix visualization software. Cell-specific transcripts were sorted using *Brain RNA-seq* database from the Barres Lab (http://www.brainrnaseq.org/). Pathway analysis and GO enrichment analyses were carried out through the use of IPA (Qiagen, Hilden, Germany) and PANTHER [47], respectively. Complete sequencing results are available in the NCBI Gene Expression Omnibus (GEO) repository and can be downloaded with the appropriate accession number.

### Statistics

**Fig. 1:**
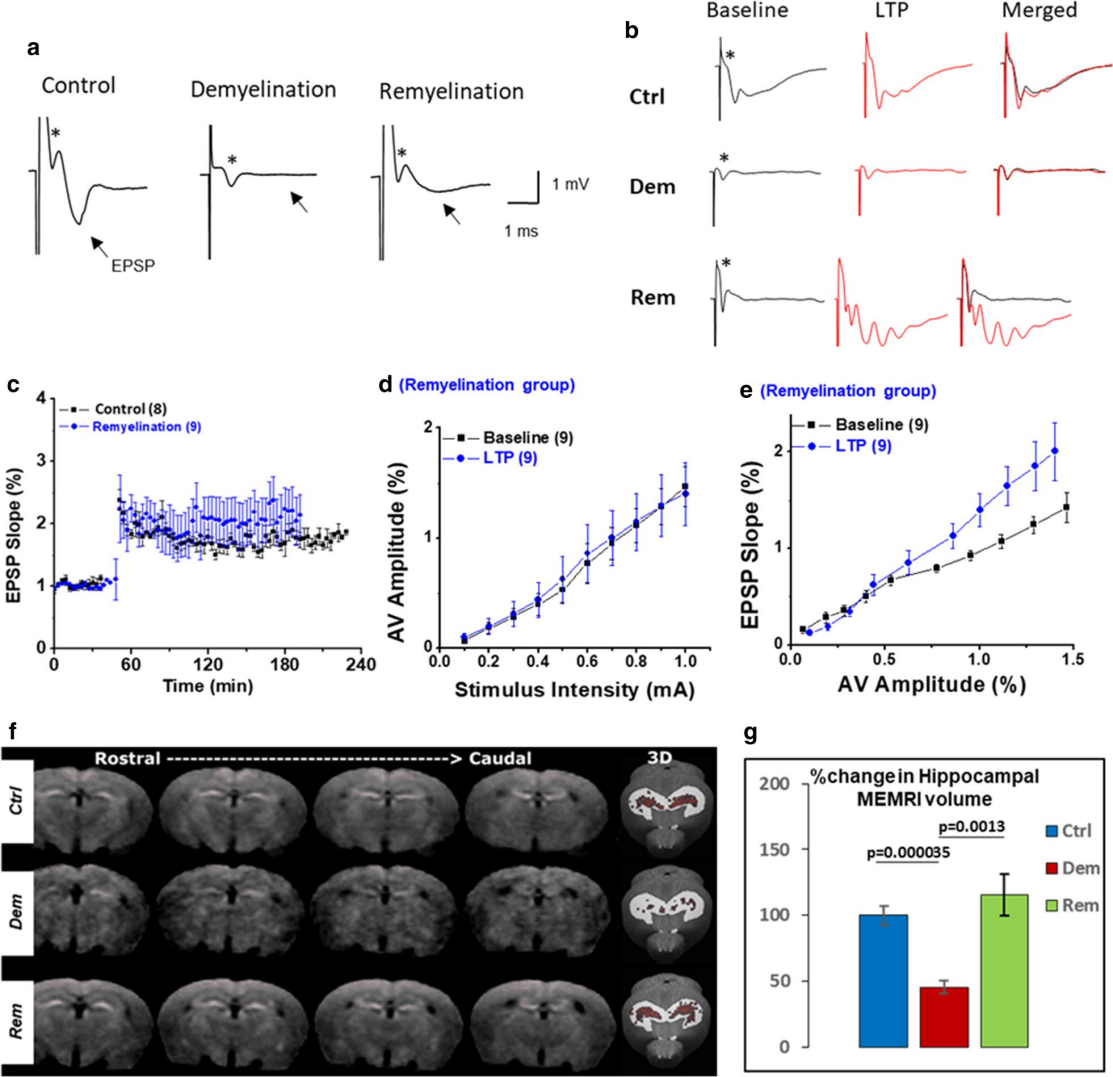
**a** Sample traces demonstrating EPSPs in hippocampal slices from control mice (left), demyelinated mice (middle, 12 weeks cuprizone), and remyelinated mice (right, 12 weeks cuprizone diet plus 6 weeks normal diet). EPSPs (arrows) present in myelinated slices were abolished by demyelination and restored by remyelination. Afferent Volley (AV) amplitude (asterisks) was similar in myelinated, demyelinated, and remyelinated slices. **b** High-frequency stimulation (HFS) (100 Hz stimulations 10 s apart) induced LTP in myelinated (Ctrl, top row) and remyelinated (Rem, bottom row) slices, but failed to induce LTP in demyelinated slices (Dem, middle row). Note multiple spikes (Rem, bottom trace) after LTP induction in remyelinated slices. **c** HFS stimulation induced comparable LTP in slices from myelinated (Control, black lines) and remyelinated slices (blue line). **d** Input–output plots indicated that HFS did not change excitability in remyelinated slices as AV amplitude remained the same across various stimulation intensity levels before and after HFS. **e** HFS induced LTP in remyelinated slices by potentiating synaptic transmission and increasing the slope of EPSPs. **f** Demyelination reduces hippocampal neuronal activity in vivo. Neuronal activity was measured in live mice using manganese-enhanced MRI. Four coronal MRI slices (the first is rostral, the fourth is caudal), in representative control (top row), demyelinated (middle row) and remyelinated (bottom row) mice, demonstrate differences in MnCl_2_-enhanced hippocampi. The rightmost image for each row is a 3D representation of MnCl_2_-enhancement in the hippocampus, with red voxels enhanced, and white voxels unenhanced. **g** Compared to myelinated hippocampi, MnCl_2_-enhanced hippocampal volume was 54% lower in demyelinated hippocampi and 7% lower in remyelinated hippocampi. Hippocampal slice studies: n indicates slice numbers. Imaging studies: n = 10 mice for each group

For analysis of in vitro hippocampal slice experiments, all means are presented ± S.E.M. and significance within a group was assessed by one-way ANOVA followed by Bonferroni’s post hoc test. Input/output plots were assessed by fitting a linear regression line using the Y = A + B * X formula to compare the slope as indicated by B. N denotes slice numbers in the text and in parenthesis in Fig. 1. For MRI experiments, the demyelinated and remyelinated groups were normalized to their respective controls by calculating the percent difference of each value per group from the mean of their controls. To evaluate differences in MRI metrics between treated and control groups, one-way ANOVA with Bonferroni’s correction for multiple comparisons tested was used. For 3D EM analyses statistical analyses were conducted with the R-statistical package and GraphPad Prism 5.0 (GraphPad Software, Inc., La Jolla, CA). All results are presented as mean ± SD except where noted. Comparisons were made by Student’s two-tailed unpaired t tests incorporating Bonferroni’s corrections for multiple comparisons where appropriate, and using F tests for variance analysis. *p* < 0.05 was considered to be statistically significant.

## Results

### Demyelination silences CA1 neuronal activity and abolishes LTP

Six weeks of cuprizone-mediated hippocampal demyelination diminishes the capacity of CA1 neurons to spontaneously fire in vivo and reduces CA1 synaptic responses in vitro [32]. The present study investigated how 12 weeks of hippocampal demyelination, as well as subsequent remyelination, alters CA1 neuronal function and LTP in hippocampal slices. We chose 12 weeks of demyelination and 6 weeks of remyelination, as they represent time points of decreased and then restored memory/learning as measure by the Morris water maze test [9]. In myelinated slices, a brief post-tetanic stimulation (PTP) of CA1 neuronal input (Schaeffer collaterals) induced an evoked response consisting of a prominent AV (Fig. 1a, Control, asterisk) followed by an EPSP (Fig. 1a, Control, arrow). EPSPs had a peak amplitude of 1.96 ± 0.24 mV (n = 16) and an average area of 4.59 ± 0.56 mV*s (n = 16). After 12 weeks of demyelination, EPSPs were completely absent (Fig. 1a, Demyelination, EPSP peak 0 ± 0, n = 20, EPSP, *p* < 0.0001 area 0 ± 0, n = 20, *p* < 0.0001); however, a prominent AV (Fig. 1a, Demyelinated, asterisk) was sustained in demyelinated slices despite complete loss of the EPSP, supporting preserved presynaptic function following demyelination. To further investigate the integrity of presynaptic function, we performed paired-pulse facilitation (PPF) experiments (Additional File 1: Fig. S1). PPF is a readout of presynaptic activity following a range (0, 50, 100, 200, 400 ms) of inter-stimulus intervals. Following the initial conditioning stimulus, the second EPSP is enhanced compared to the prior stimulus [48] (Additional File 1: Fig. S1a). The PPF response was maintained in hippocampal slices obtained from mice on the cuprizone diet for 1 (n = 4), 3 (n = 4), or 4 weeks (n = 4) (Additional File 1:Fig. S1b). In light of the preserved AVs in the absence of EPSPs in hippocampal slices demyelinated for 12 weeks (Fig. 1a), these PPF data provide additional support for the concept that presynaptic function of Schaeffer collaterals is maintained during hippocampal demyelination (Additional File 1: Fig. S1a). In addition, presynaptic function, as measured by PPF, was similar between control, demyelinated, and remyelinated slices (Additional File 1: Fig. S1a, c), indicating that presynaptic function of CA1 synapses was not affected by the demyelination/remyelination processes. Schaeffer collateral axons are not myelinated, and this may play a role in their preserved function following demyelination. In summary, 12 weeks of hippocampal demyelination completely abolished CA1 neuronal EPSPs, despite sustained presynaptic activity. Six weeks of remyelination partially restored CA1 neuronal EPSPs.

Since LTP is a widely-accepted readout of memory/learning [28], we investigated the effects of hippocampal demyelination and remyelination on LTP. LTP was induced in myelinated slices by high-frequency stimulation (HFS) of Schaeffer collaterals (Fig. 1b: traces show baseline, HFS, and merged baseline/HFS). HFS induced LTP in myelinated slices (Fig. 1b, top row), but failed to induce LTP in demyelinated slices (Fig. 1b, middle row, baseline vs. LTP traces). In remyelinated slices, LTP was restored to levels comparable to myelinated slices (Fig. 1b; lower row). LTP in remyelinated slices was similar in magnitude and duration (Fig. 1c, blue circles) to LTP in slices obtained from control animals (Fig. 1c, black squares). Restoration of LTP after remyelination was not due to a significant change in presynaptic excitability, as AV amplitudes at various stimulation strengths were identical at baseline in the remyelinated slices (Fig. 1d, 1.43 ± 0.09 vs 1.42 ± 0.16). The observed LTP correlated with an increase in synaptic output, which was greater following HFS compared to baseline in remyelinated slices (Fig. 1e; 1.37 ± 0.08 blue squares vs 0.87 ± 0.07 black squares, 154% increase in synaptic transmission, n = 9, *p* < 0.0002). These studies establish that 12 weeks of hippocampal demyelination reversibly abolishes CA1 neuronal activity and LTP.

### Demyelination decreases hippocampal neuronal activity in vivo

To extend our acute slice studies to living mice, we examined whether hippocampal demyelination and remyelination altered neuronal firing using manganese-enhanced MRI. Hippocampal volumes were also measured from structural MRI. Compared to myelinated hippocampal volume, analysis of structural MRI detected a significant decrease (11.1%) in hippocampal volume following demyelination (Additional File 1: Fig. S2). Hippocampal volume loss was not restored by remyelination (Additional File 1: Fig. S2). MnCl_2_-enhanced hippocampal volume is shown in Fig. 1f. Four coronal MRI slices (the first is rostral, the fourth is caudal), in representative control (top row), demyelinated (middle row) and remyelinated (bottom row) mice, demonstrate differences in MnCl_2_-enhanced hippocampi. The rightmost image for each row is a 3D representation of MnCl_2_-enhancement in the hippocampus, with red voxels enhanced, and white voxels unenhanced. Compared to myelinated hippocampi, MnCl_2_-enhanced hippocampal volume was 54% lower (*p* < 0.00001) in demyelinated hippocampi (Fig. 1g) and 7% higher in remyelinated hippocampi (Fig. 1g). These results support reduced neuronal firing following 12 weeks of hippocampal demyelination in living mice and establish that reduced hippocampal volume does not significantly contribute to reduced MnCl_2_-enhanced hippocampal volume in demyelinated hippocampi. Thus, neuronal firing (Fig. 1g), but not hippocampal volume (Additional File 1: Fig. S2), was restored by remyelination.

### Hippocampal demyelination alters gene transcripts associated with myelination and innate immunity

**Fig. 2:**
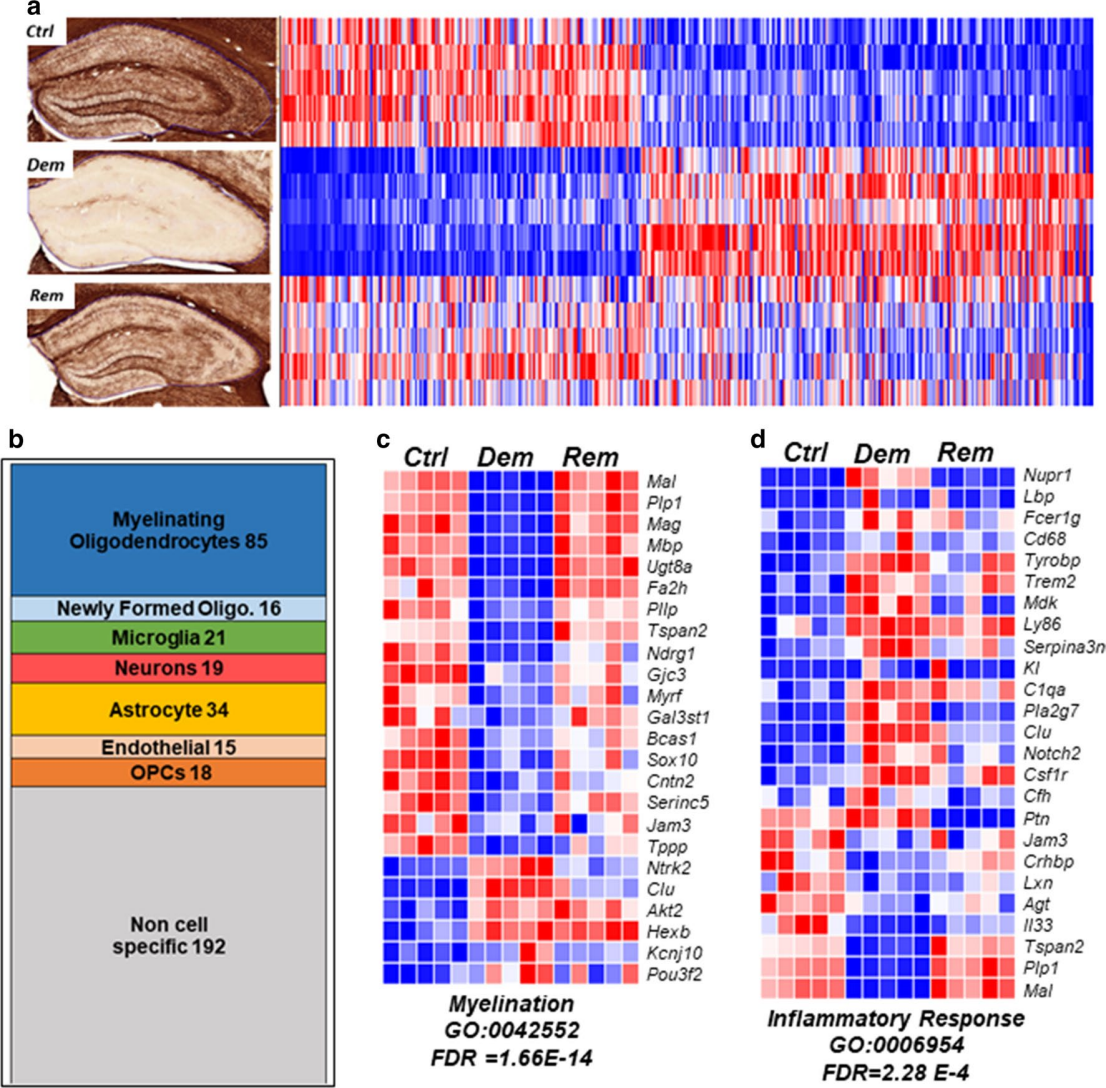
Demyelination alters 400 hippocampal gene transcripts. **a** Left panels show myelin content of myelinated (Ctrl), demyelinated (Dem), and remyelinated (Rem) hippocampi. Right panels show heat maps of differentially-expressed gene transcripts in the three conditions (n = 5). Four-hundred gene transcripts were significantly altered by demyelination (*p* < 0.05 FDR), and a majority of these trended toward control levels following remyelination. High expression levels are shown in red and lower expression levels are in blue. **b** Two-hundred and eight transcripts were CNS cell-enriched, while 192 were ubiquitously expressed by CNS cells. CNS cell-enriched transcripts can be found in Additional File 1: Fig. S2 and S3. **c** Based upon gene ontology (GO) analysis of biological processes, transcripts associated with “Myelination” were significantly altered by demyelination. Transcripts encoding myelin proteins were significantly decreased. **d** GO enrichment analyses of the biological process “Inflammatory Response” identified altered transcripts associated with innate immunity. In contrast to decreased myelin transcripts, many immune response transcripts were increased by demyelination

We used RNA sequencing to compare gene transcripts in myelinated (n = 5), demyelinated (n = 5), and remyelinated (n = 5) hippocampi (Fig. 2a). Compared to myelinated hippocampus, 400 gene transcripts were significantly altered (FDR adjusted < 0.05) following 12 weeks of demyelination (Fig. 2a). Following 6 weeks of remyelination, most of these transcripts returned to or trended towards transcript levels found in myelinated hippocampus (Fig. 2a). Using the brain cell-specific *RNA-seq* database [49], these 400 transcripts were sorted for cell enrichment. Of the 400 altered transcripts, 19 encoded proteins were enriched in neurons, 21 in microglia, 34 in astrocytes, 85 in oligodendrocytes, 16 in newly formed oligodendrocytes, 18 in oligodendrocyte progenitor cells, 15 in endothelial cells, and 192 were expressed by multiple CNS cell types (Fig. 2b; gene lists and a volcano plot of select DEGs are shown in Additional File 1: Fig. S5).

We then performed GO enrichment for biological processes of interest. As expected, the most altered biological process was myelination (Fig. 2c, FDR = 1.66E−14. GO.0042552). Eighteen transcripts, including those that encode myelin proteins (PLP, MBP, MAG, and MAL) and proteins involved in lipid biosynthesis (UGT8a, Fa2h), were significantly reduced in demyelinated hippocampi and returned to control levels in remyelinated hippocampi. These data provide a signature of oligodendrocyte transcript changes for this cuprizone model of hippocampal demyelination and remyelination.

### Inflammatory Response in demyelinated hippocampi

Cuprizone is a toxin that kills oligodendrocytes. While the peripheral immune system may be involved in the removal of myelin debris, it is not required for cuprizone-mediated demyelination [50]. Staining for the pan microglial marker, IBA1, did not detect hypertrophy or increased microglial density in hippocampi demyelinated by cuprizone for 12 weeks [33]. The GO inflammatory response transcript profile (Fig. 2d, FDR = 2.28E−4. GO.0006954), however, implicates innate immunity as a regulator of neuronal integrity in the demyelinated hippocampus. Of the 17 immune-related transcripts increased by 12 weeks of demyelination, 9 are expressed by hippocampal microglia and associated with innate immune responses. TREM2 [51] and its activator Tyrobp [52], CSF1R [53], and KL [54] promote neuronal protection. PTN and its activator MDK [55] suppress LTP and apoptosis. Pla2g7 positively correlates with cognitive dysfunction [56], while Clu and Serpina3n are negatively associated with neurodegenerative diseases [57].

### Hippocampal demyelination does not alter dendritic spines of CA1 neurons

**Fig. 3:**
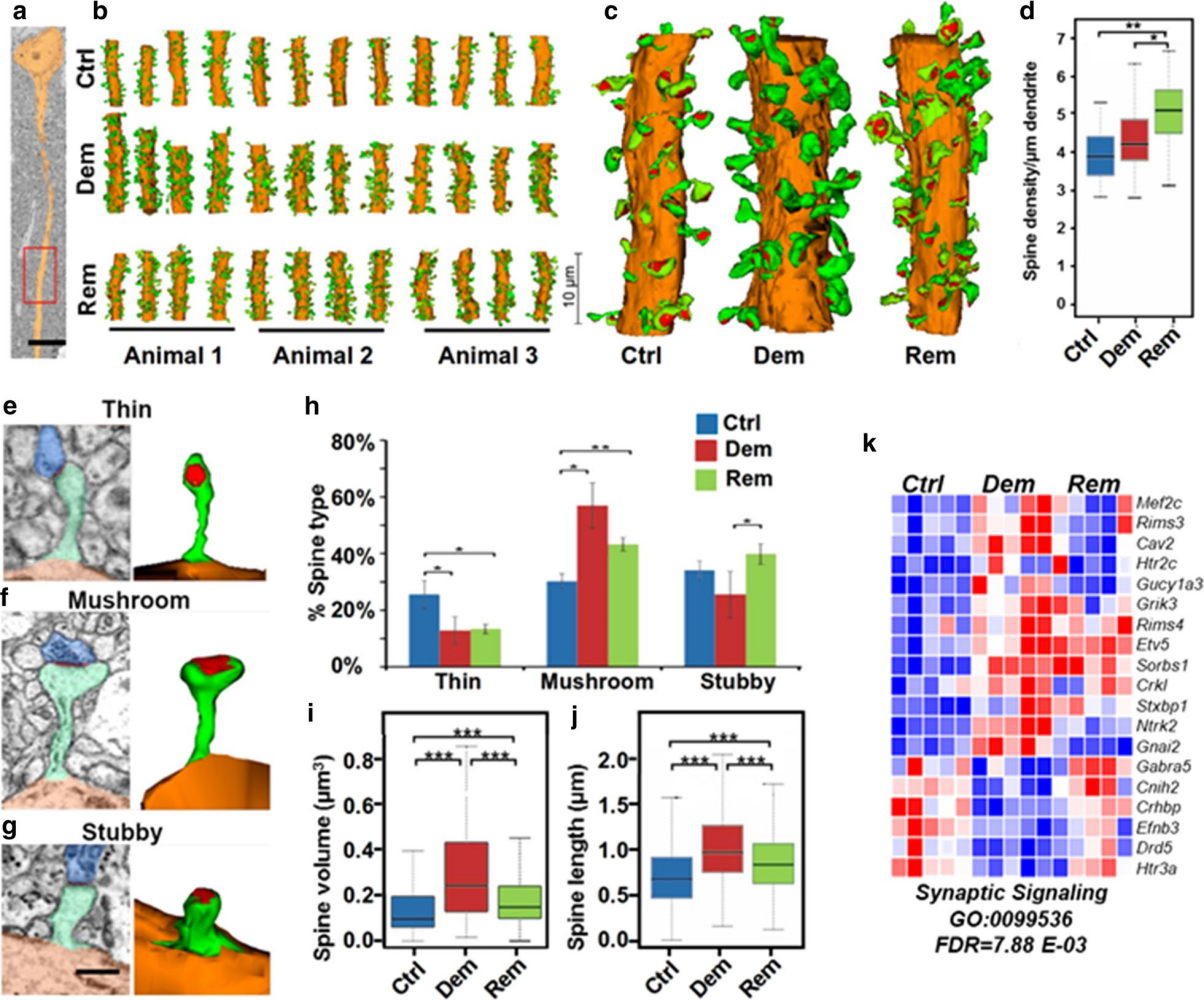
Dendritic spines of CA1 neurons are not decreased nor ultrastructurally altered by demyelination. We used 3D EM to characterize the dendritic spines of CA1 neurons. **a** Low magnification of a CA1 neuron and its primary dendrite. Box shows an area of spine analyses that corresponds to regions where electrophysiology studies were performed in acute slices. **b** Three-dimensional reconstructions of dendritic spines from myelinated (Ctr), demyelinated (Dem), and remyelinated (Rem) hippocampi. Four reconstructed dendrites from 3 mice were created for each condition. Each reconstruction included 300–500 serial, 75 nm-thick sections captured at 5–7 nm per pixel resolution. **c** Higher magnification of 3D reconstructions showing spine shape (green) and post-synaptic densities (red). **d** Spine density was similar in myelinated and demyelinated hippocampi and significantly increased in remyelinated hippocampi. **e–g** Based upon shape, spines were divided into three categories (thin, mushroom and stubby). **h** The percentage of the structurally more mature mushroom-shaped spines was significantly increased in demyelinated and remyelinated hippocampi, while less mature thin spines were decreased. **i-j** Compared to myelinated hippocampi, total spine volume and spine length were increased in demyelinated hippocampi. Compared to demyelinated hippocampi, remyelinated hippocampi had smaller spine volumes and lengths. **k** RNA-seq analysis identified alterations in 19 Gene Ontology synaptic signaling transcripts. See the Results section for possible roles of protein encoded by these transcripts in reduced synaptic function

The silencing of EPSPs with intact AVs following demyelination (Fig. 1a) implies dysfunction of dendritic spines in CA1 neurons. We used 3D EM to investigate the dendritic spines that facilitate physiological changes described in the acute slices. The box in Fig. 3a shows the dendritic region of a CA1 neuron that was analyzed by 3D EM. Figure 3b, c show reconstructed spines from myelinated, demyelinated, and remyelinated hippocampi. Surprisingly, the density of dendritic spines was similar in myelinated and demyelinated hippocampi and averaged between 4 and 4.5 spines/μm spine length (Fig. 3d). Compared to myelinated and demyelinated hippocampi, the density of dendritic spines was significantly increased following six weeks of remyelination (Fig. 3d). Criteria reported by Harris and colleagues [58] were used to characterize individual dendritic spines based upon shape: thin (Fig. 3e), mushroom (Fig. 3f), or stubby (Fig. 3g). Mushroom-shaped spines represent mature and physiologically active excitatory synapses, while stubby and thin spines reflect less mature and less active excitatory synapses [59]. Compared to myelinated hippocampus, a 40% increase in mushroom-shaped dendritic spines was found in demyelinated hippocampus (Fig. 3h). This increase was at the expense of thin spines, which were reduced by over 50% (Fig. 3h). Compared to myelinated hippocampi, mushroom-shaped spines were increased in remyelinated hippocampi (Fig. 3h), but the increase in mushroom-shaped spines was less than that found following 12 weeks of demyelination (Fig. 3h). Total spine volume (Fig. 3i) and spine length (Fig. 3j) were significantly increased in demyelinated and remyelinated hippocampi, and were greater in demyelinated than in remyelinated hippocampi. Filamentous spines were included in our quantitative analyses, but showed no difference between groups and are not included in Fig. 3. In summary, demyelination increases the ultrastructural maturation and volume of CA1 dendritic spines.

### Hippocampal demyelination alters gene transcripts that encode synaptic signaling proteins

Compared to myelinated hippocampi, nineteen GO synaptic signaling transcripts were altered in demyelinated hippocampi (Fig. 3k, FDR = 7.88E−03. GO:0099536) and most returned toward control levels in remyelinated hippocampi. These altered transcripts are expressed by neurons. Six transcripts were decreased by demyelination, and reductions in five of these have been associated with decreased neuronal activity (Htr3A [60], CNIH2 [61], loss of LTP (Drd5 [62] and Efnb3 [63], and altered cognitive function (Htr3A [60] Drd5 [62], Efnb3 [63], CNIH2 [61], and GABARa5 [64]). The thirteen transcripts increased by demyelination are associated with a variety of neuronal functions including gene transcription (ETV5 [65] and MEF2c [66], neurotransmitter receptors (NTRK2 [67], GRIK3 [68], and HTR2C [69]), synaptic vesicle docking (Stxbp1 [70], RIMS4 and RIMS3 [71;72]), G protein signaling (GNAI2 [73] and CRK1 [74]) and dendritic development (ETV5 [65] and CRK1 [74]).

### Demyelination increases the volume and shape of PSDs

**Fig. 4:**
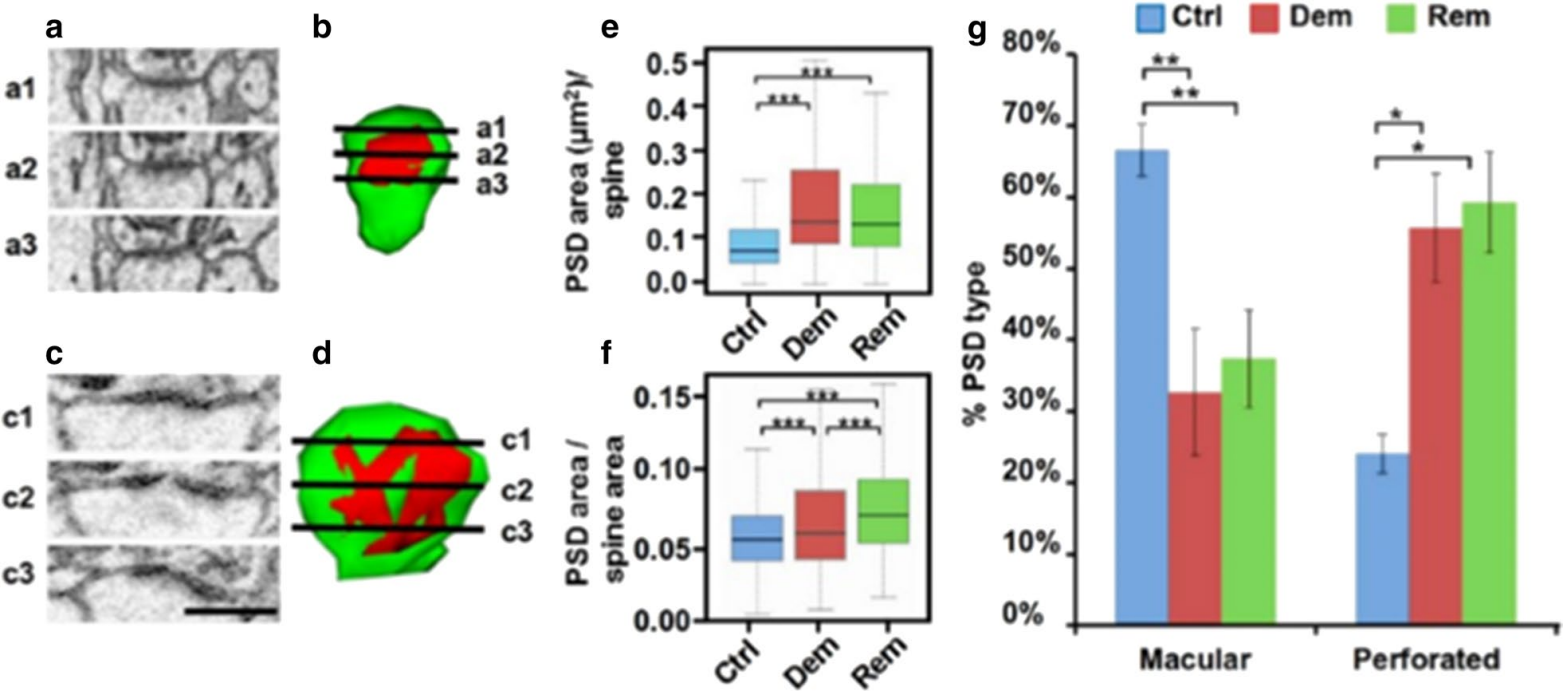
Post-synaptic density (PSD) area is increased and appears more mature in demyelinated hippocampi. We reconstructed the size (area) and shape of PSDs in serial 3D EM reconstructions. **a** Three EM images of spine head showing PSD beneath post-synaptic membrane. **b** Reconstruction of this macular-shaped spine showing orientation of the images in panel a (a1-a3). **c** Three serial EM images of spine head showing PSD beneath post-synaptic membrane. **d** Reconstruction of this perforated-shaped spine showing orientation of the images in panel c (c1-c3). **e** Compared to myelinated hippocampi, PSD area/spine is significantly increased in demyelinated and remyelinated hippocampi. **f** After correcting for the increase in total spine area, PSD area was still increased in demyelinated and remyelinated hippocampi. **g** The percentage of perforated PSDs was significantly increased in demyelinated and remyelinated hippocampi

The active zones of excitatory dendritic spines have a specialized PSD that concentrates glutamate receptors and a host of signaling and scaffolding molecules that are essential for normal synaptic function [75]. While most PSDs have a disc-like or macular shape, PSDs with a perforated shape are considered to have a greater concentration of glutamate receptors and to be more common in larger, more mature mushroom-shaped spines [75]. PSDs were identified in serial EM sections (Fig. 4a, c) and reconstructed in 3D (Fig. 4b, d). Total PSD area was significantly greater in demyelinated and remyelinated hippocampi when compared to myelinated hippocampi (Fig. 4e). Since spine volume was increased in myelinated and demyelinated hippocampi (Fig. 3i), we measured total PSD area based upon total spine area. Total PSD area was increased after correcting for total spine head area (Fig. 4f), identifying a PSD increase independent of spine head volume increase. In myelinated hippocampi, the percentage of macular-shaped spines was twice that of perforated spines (Fig. 4g). This ratio was reversed in demyelinated and remyelinated hippocampi (Fig. 4g). In summary, PSD volumes in demyelinated hippocampi are increased and structurally appear more mature. Our ultrastructural studies therefore indicate that the function of dendritic spines cannot be judged solely by spine shape and PSD ultrastructure.

### Hippocampal demyelination increases astrocyte participation in the tripartite synapse

**Fig. 5:**
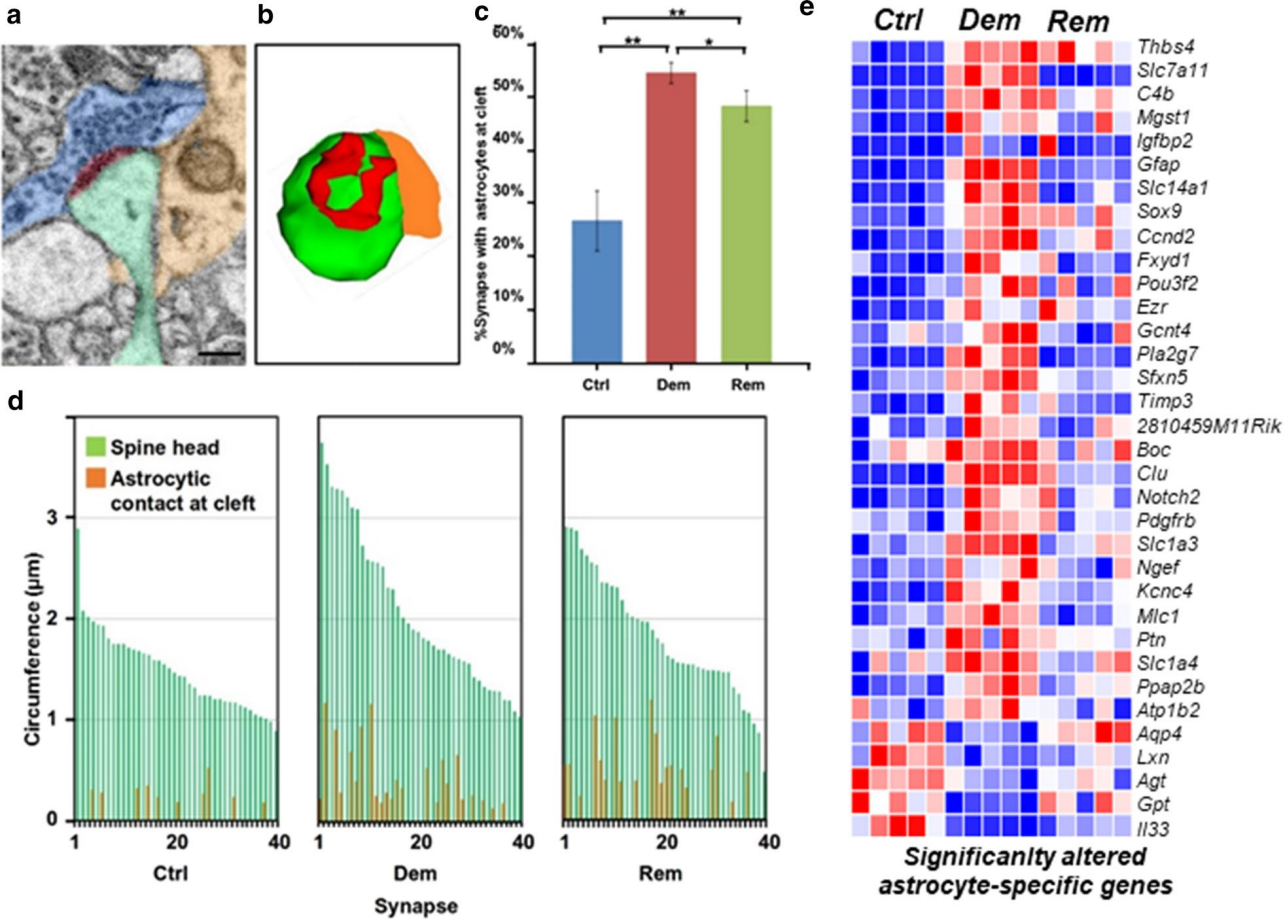
Demyelination increases astrocyte participation in the tripartite synapse. **a** EM image showing presynaptic terminal (blue), dendritic spine (green), and astrocyte process apposing synaptic cleft (tan). **b** Reconstruction of the relationship between the astrocyte and the synaptic terminal. **c** Compared to myelinated hippocampi, the percentage of synapses with astrocytes at the synaptic cleft is significantly increased in demyelinated and remyelinated hippocampi. **d** Correlation between spine head volume (green lines) and astrocyte participation (tan lines) in the tripartite synapse. Compared to myelinated hippocampi, demyelinated and remyelinated hippocampi have increased astrocyte participation in the tripartite synapse that is independent of spine head volume. Demyelination increased the percent increase in astrocyte contact. **e** RNA-seq analysis identified alterations in 29 astrocyte-specific transcripts in demyelinated hippocampus. Of note are increases in transcripts that encode astrocyte proteins that reduce extracellular glutamate (Slc7a11) and suppress CA1 neuronal activity (Slc1A3)

Astrocytes, the third component of the tripartite synapse, play an important role in excitatory neurotransmission [76–78]. Two overlapping roles for the perisynaptic astrocyte have been proposed. They may directly participate in neurotransmission by release of glutamate [78], and they also provide a structural barrier that isolates individuals synapses and prevents spill in and spill out of glutamate [79]. Reductions in astrocyte coverage of CA1 synapses prolong EPSPs and increase mGluR activation [80]. Here, we compare astrocyte participation in CA1 tripartite synapses in myelinated, demyelinated, and remyelinated hippocampi using 3D EM reconstructions (Fig. 5a, b). If the cell source of a perisynaptic cellular process was not apparent, then the process was followed into the parenchyma. Astrocytic origin was confirmed by the presence of intermediate filaments and/or glycogen granules, which are two ultrastructural hallmarks of astrocytes [81]. Compared to myelinated hippocampi, the number of synaptic clefts covered by astrocyte processes doubled in demyelinated and remyelinated hippocampi (Fig. 5c). As synaptic spine head circumference increased in demyelinated hippocampi, the extent of astrocyte contact with the synaptic cleft also increased (Fig. 5d). These data establish that demyelination increases astrocyte participation in the tripartite synapse.

### Hippocampal demyelination alters gene transcripts that encode astrocyte proteins

We next queried astrocyte-specific transcripts that were significantly changed in demyelinated hippocampi. Twenty-nine transcripts were increased and 5 were decreased following 12 weeks of cuprizone demyelination (Fig. 5e). Four increased transcripts (GFAP, Slc7a11, Slc1A3, and Clu) encode proteins of interest. GFAP encodes the astrocyte intermediate filament protein, GFAP, which can be a marker for astrocytosis. Despite the increase in GFAP transcripts, the hippocampal area occupied by GFAP is not increased by 12 weeks of demyelination [33]. Slc7a11 and Slc1A3 encode antiporters that are enriched in astrocyte processes that participate in the tripartite synapse [82, 83]. Slc7a11 encodes xCt, a glial antiporter, which exports glutamate and imports cysteine and has been shown to suppress glutamergic synaptic strength of CA1 neurons [84]. Slc1A3 encodes the antiporter EAAT1/GLAST1, which can reduce extracellular glutamate and neuronal toxicity [85, 86] and thereby help maintain the integrity of the tripartite synapse and CA1 neuron. Clu encodes clusterin, a member of the heat shock protein family that protects neurons from apoptosis [87]. GWAS studies have also identified Clu as risk factor for Alzheimer’s and Parkinson’s diseases [88]. Increased astrocyte participation in the tripartite synapse and their increased expression of molecules that harness extracellular glutamate support a role for astrocytes in dendritic spine silencing and stabilization of synaptic integrity in demyelinated hippocampi.

## Discussion

LTP in hippocampal CA1 pyramidal neurons is a major cellular mechanism that underlies learning and memory [89]. Cognitive dysfunction and hippocampal demyelination are common features in individuals with the demyelinating disease MS [90]. The present study investigated physiological, ultrastructural, and gene transcript changes following demyelination and remyelination of the hippocampus in a rodent model of MS. Hippocampal demyelination abolished LTP and EPSPs of CA1 pyramidal neurons in acute slices. Using in vivo MEMRI, we showed that hippocampal demyelination reduced CA1 neuronal activity in live mice without compromising the density or ultrastructural integrity of their dendritic spines. Four hundred hippocampal gene transcripts were significantly altered by demyelination. Neuronal gene transcript changes are consistent with dendritic spine silencing. Microglia and astrocytes also participate in spine silencing and appear to be programmed to protect the tripartite synapse and the CA1 neuron. We postulate that CA1 neurons survive demyelination and hibernate in a state that protects the demyelinated axon and facilitates functional recovery following remyelination. Remyelination partially/totally rescued the changes found in demyelinated hippocampi. Remyelination therapies, therefore, could have an impact on hippocampal and cognitive function in MS.

Neurological disability associated with new white-matter demyelination is thought to be caused by conduction block at the site of demyelination [91]. Our data establish that neuronal dysfunction associated with hippocampal demyelination can also be mediated at the dendritic spines of CA1 neurons that project demyelinated axons. Why would CA1 neurons with demyelinated axons silence their dendritic spines? Demyelination results in a redistribution of Na^+^ channels from nodal axolemma to all regions of the demyelinated axolemma. This dramatically increases Na^+^ influx during nerve conduction and increases the energy demands for exchanging axoplasmic Na^+^ for extracellular K^+^ [91]. Failure to exchange axonal Na^+^ for extracellular K^+^ will activate the Na^+^/Ca^2+^ exchanger and dramatically increase axoplasmic concentrations of Ca^2+^, which can induce a virtual axonal hypoxia and cause axonal degeneration [91]. While the axon eventually compensates for this increased energy demand by increasing the volume of axoplasmic mitochondria [92–95], it may be initially vulnerable to the increased ionic exchange associated with loss of myelin. We consider dendritic silencing to be a neuroprotective response that reduces axonal conduction and helps prevent degeneration of demyelinated axons.

Surprisingly, the dysfunctional dendritic spines appear to be ultrastructurally more mature, with increased mushroom shape and increased perforated PSDs. Mushroom-shaped spines are stable for months [96] and provide a structural basis for long-term memory [97]. Increased maturation of dendritic spines following demyelination may facilitate functional recovery following remyelination, and thus helps re-establish LTP. While microglial-mediated synaptic pruning can cause memory loss [98], we observed no loss of dendritic spines projected by CA1 neurons in demyelinated hippocampus. It remains to be determined how long ultrastructurally-mature dendritic spines are maintained in more chronic hippocampal lesions. Postmortem studies of demyelinated hippocampi obtained from end-stage MS patients reported significant decreases in synaptic densities and decreased expression of neuronal genes associated with axonal transport, glutamate neurotransmission, glutamate homeostasis, and memory/learning [9]. It is likely that these hippocampi were demyelinated for decades. Our attempts to prolong cuprizone demyelination beyond 12 weeks have not been successful. Development of rodent models with more prolonged hippocampal demyelination are needed to fill the gap between the rodent studies described here and the published studies of demyelinated human hippocampi.

How do CA1 neurons silence their dendritic spines? While our molecular studies failed to identify a “silver bullet”, they provide important clues regarding mechanisms. Gene transcripts encoding glutamate receptors were not significantly reduced. Recent studies support the possibility that trafficking and nanoscale positioning of glutamate receptors in dendritic spine surface membranes, rather than absolute numbers of glutamate receptors, regulate the efficacy of synaptic transmission in both normal and disease states [99]. Glutamate receptor positioning and post-translational modifications of glutamate receptors are plausible mechanisms of CA1 dendritic spine silencing. When synaptic signaling transcripts were probed, several candidates known to decrease neuronal firing and contribute to cognitive decline were altered (Fig. 3k). Future studies are needed to delineate any direct role they may have in dendritic spine silencing.

Astrocytes and microglia modulate the brain microenvironment and have been associated with protective and destructive roles in demyelinating diseases. During demyelination of the corpus callosum, astrocyte-mediated microglial activation enhances myelin debris removal and facilitates remyelination [100]. Following 12 weeks of cuprizone demyelination, hippocampal areas occupied by GFAP-positive astrocytes and Iba1-positive microglia are identical to those found in myelinated hippocampus [33]. Gene transcript analyses, however, indicate that astrocytes and microglia are activated. In contrast to decreased myelin transcripts (Fig. 2c), 29/34 astrocytic transcripts (Fig. 2d) and 17/25 microglial transcripts (Fig. 2d) were increased following 12 weeks of hippocampal demyelination, while synaptic signaling transcripts were increased and decreased (Additional File 1: Fig. S5). Astrocyte processes also significantly increased their participation in the tripartite synapses (Fig. 5c) and increased transcripts that encode antiporters, which suppress glutamatergic synaptic strength [84] and harness extracellular glutamate (Fig. 5e). Microglia increased transcripts associated with neuronal protection and suppression of LTP (Fig. 2d). Microglia also remove synapses during development [101] and disease [45, 102–104]. Reductions in synapse number and microglial stripping of CA1 synapses were not apparent in our 3D EM studies. Astrocytes and microglia protect CA1 neurons and may play a role in silencing their synaptic activity.

There are limitations to this study. Since a comprehensive rodent model of MS does not exist, we are limited to modeling specific aspects of the disease. The only rodent model that causes reproducible, prolonged, and near complete hippocampal demyelination is the cuprizone model. This demyelination does not require participation of peripheral immune cells, so the active demyelinating stage differs from hippocampal demyelination in individuals with MS. Recent studies have reported microglia-mediated synaptic pruning in the visual thalamus of postmortem MS brains and in immune-mediated animal models of MS [105]. Since MS patients rarely die during acute stages of the disease, demyelinated hippocampi at time points similar to those used in the present study are rarely available. While our data support retention of presynaptic CA1 activity of Schaeffer collaterals when stimulated in vitro, it remains to be established if Schaeffer collaterals are active in the demyelinated hippocampus in vivo. There are also limitations to our transcript profiling studies that were obtained from whole hippocampi. Future studies are needed to unravel the molecular mechanisms of CA1 dendritic spine silencing in demyelinated hippocampus. The publicly-available transcriptome database accompanying this manuscript provides a platform for generating and testing hypotheses of dendritic silencing that involve neurons, oligodendrocytes, astrocytes, and microglia.

## Supplementary Information

**Additional file 1: Fig. 1** Presynaptic activity of Schaeffer collaterals is maintained during demyelination and following remyelination **Fig. 2** Hippocampi were segmented from T2w MRI and their volumes were quantified **Fig. 3** CNS cell-specific transcripts that were significantly altered by demyelination and partially restored by remyelination **Fig. 4** CNS cell-specific transcripts that were significantly altered by demyelination and partially restored by remyelination **Fig. 5** Volcano plot of selected gene transcripts that are significantly altered in demyelinated hippocampi

**Additional file 2.** Serial EM sections used for dendritic spine reconstructions. Dendritic spines are colored yellow. Presynaptic terminals are colored blue

**Additional file 3.** Video showing reconstruction of spines projecting from a dendrite
