# Supplementary material for: Neuronal hibernation following hippocampal demyelination
Source: Acta Neuropathol Commun. 2021 Mar 1;9:34. doi: 10.1186/s40478-021-01130-9 (PMC7923530; doi:10.1186/s40478-021-01130-9)
Supplement: Supplementary file 1 — Additional file 1: Fig. 1 Presynaptic activity of Schaeffer collaterals is maintained during demyelination and following remyelination Fig. 2 Hippocampi were segmented from T2w MRI and their volumes were quantified Fig. 3 CNS cell-specific transcripts that were significantly altered by demyelination and partially restored by remyelination Fig. 4 CNS cell-specific transcripts that were significantly altered by demyelination and partially restored by remyelination Fig. 5 Volcano plot of selected gene transcripts that are significantly altered in demyelinated hippocampi [file 40478_2021_1130_MOESM1_ESM.pdf]

Supplemental Figure 1

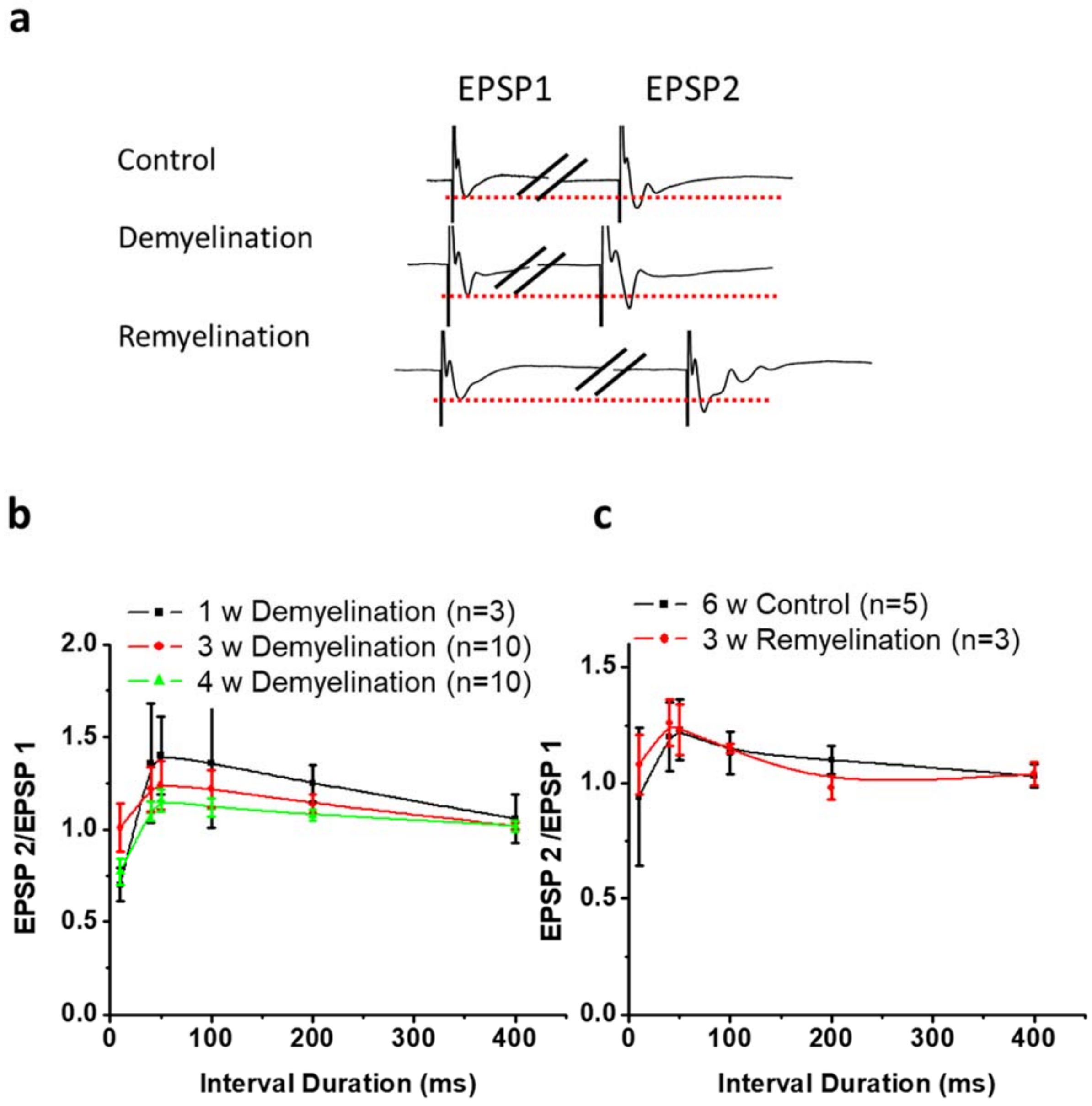

Supplemental Fig. 1 Presynaptic activity of Schaeffer collaterals is maintained during demyelination and following remyelination. **a** Sample traces show a prominent increase in the second EPSP (EPSP2) amplitude evoked using a Paired-Pulse Facilitation (PPF) paradigm with 50 ms intervals in control slices (age-matched to 4 weeks), demyelinated slices (4 weeks), and slices demyelinated for 6 weeks and remyelinated for 3 weeks. The PPF ratio is quantified as the ratio of the amplitude of EPSP2 over the first EPSP (EPSP1) over a range of intervals (0, 50, 100, 200, 400 ms), and is shown for **b** demyelinated slices (1, 3, and 4 weeks), and **c** comparing remyelinated (6 weeks demyelination + 3 weeks remyelination) and control slices (age-matched). n indicates the number of slices.

Supplemental Figure 2

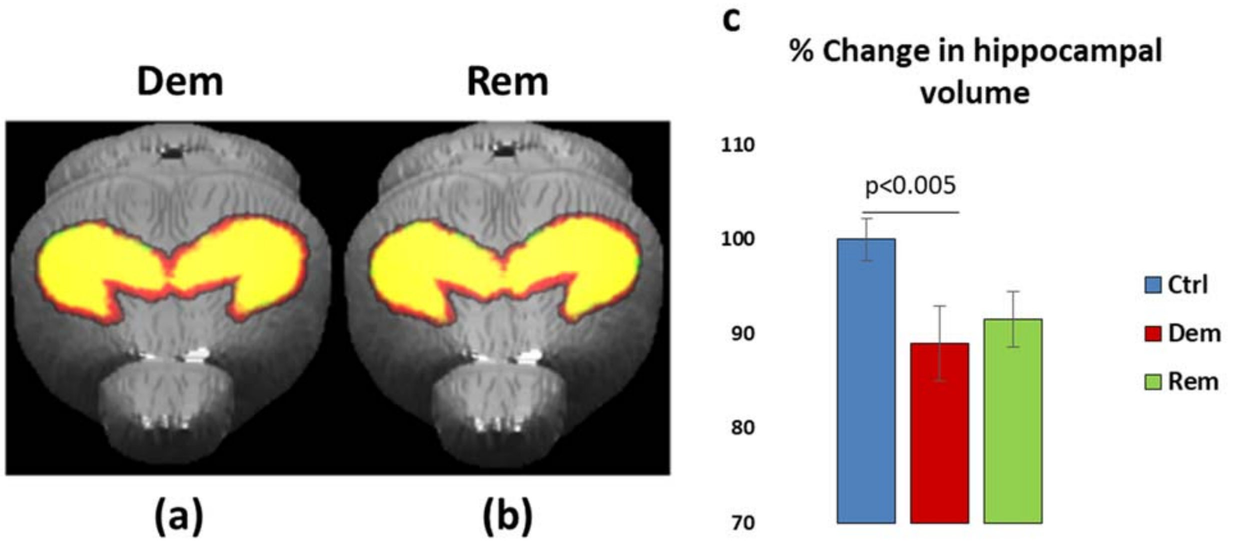

Supplemental Fig. 2 Hippocampi were segmented from T2w MRI and their volumes were quantified. Hippocampi after demyelination **(a)** and after remyelination **(b)** are shown in yellow. **c** Compared to myelinated hippocampi, hippocampal volumes after demyelination were reduced on average by 11.1%. Hiippocampal volumes after remyelination were not significantly different from hippocampal volumes after demyelination. n= 10 mice per group.

Supplemental Figure 3

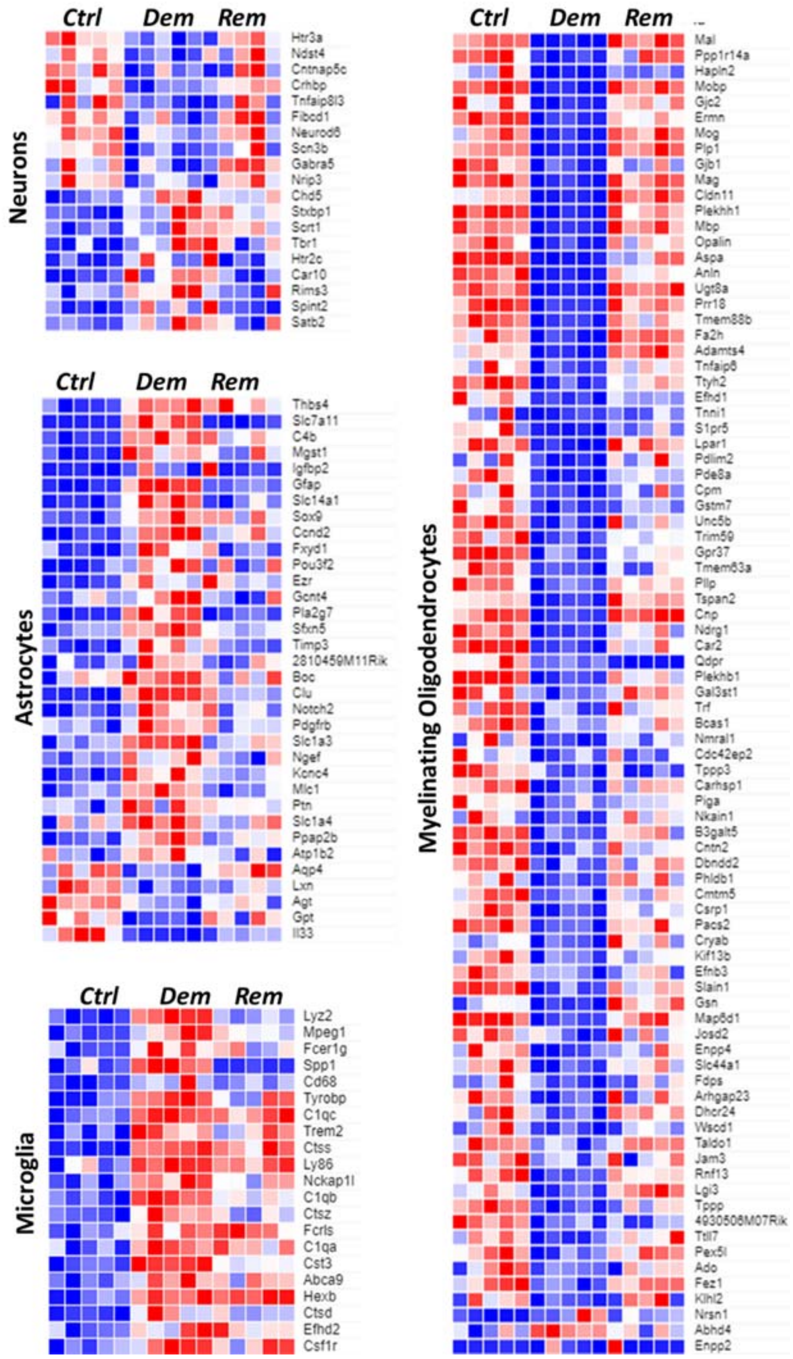

Supplemental Fig. 3 CNS cell-specific transcripts that were significantly altered by demyelination and partially restored by remyelination. Data for neurons, astrocytes, microglia, and myelinating oligodendrocytes are shown in the heatmaps. Higher expression levels are indicated in red and lower expression levels are indicated in blue. Complete sequencing results are available in the NCBI Gene Expression Omnibus (GEO) repository and can be downloaded with the appropriate accession number.

Supplemental Figure 4

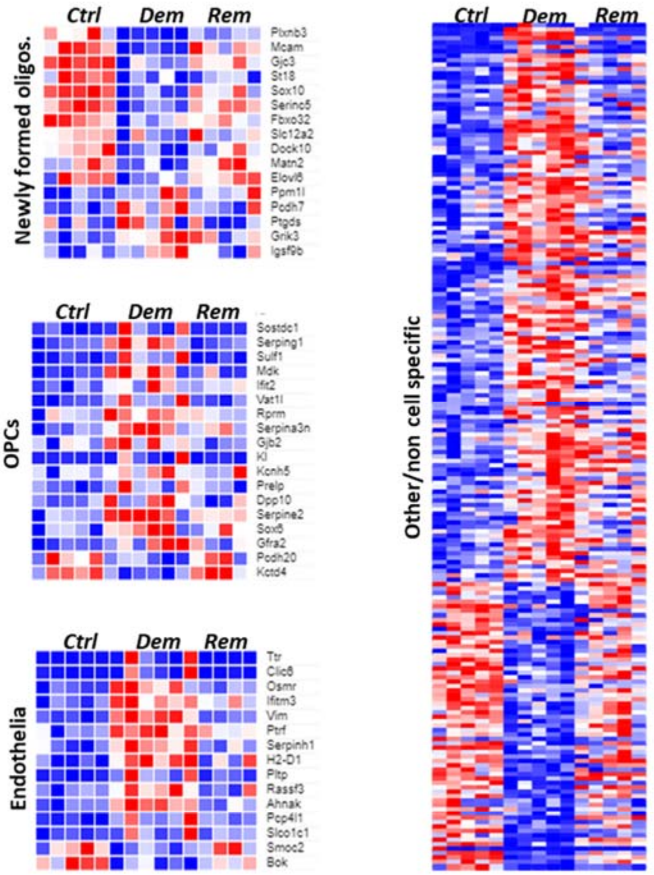

Other/non cell specific gene list

Aqp1	Efemp2	Hrsp12	B2m	Akt2	Ogdhl	Hlf	Epas1	Iqgap2	Bin1	Gm2115	Mir6240
Nupr1	Etnppl	Tmem176b	Sat1	Igfbp5	Cebpg	Sorbs1	Prkaa2	Tspan13	Commd3	Lrrc10b	Mir6236
Atf5	Nelfe	Cpq	Antxr1	Cdh18	Gpr37l1	Map3k9	Ntrk2	Sparc	Gstm5	Gm22513	Alpk2
Ggta1	Coch	Tgfbr2	Utrn	Cnn3	Ldhb	Mtss1l	Kcnj9	Gstm1	Prox1	Pkp2	Gm15662
Lbp	Lamp5	Cav2	Zfp36l2	Itpkb	Ubald1	Anp32e	Capn2	Tubb2a	Lrrn1	Erbb2ip	Insig1
Cdkn1a	Gm11549	Mbd3	Adra1a	Pacsin2	Sft2d2	Ift172	Rps6ka2	Nbas	Dynll1	Plcl1	Tbata
Ace	Rhbdd1	Fnbp1l	Trpm3	Camk2d	Hbb-bs	St3gal5	Mark1	Golm1	Adipor2	Slc9a2	Myrf
Ptprc	Luzp2	Slc38a3	Kcnj10	Hba-a1	Sesn3	Crkl	Gnai2	Map7	Nr3c2	Hmgcs1	Gatm
Ifit3	Apoe	Lhfp	Tmem47	42624	Ptprm	Eif3b	Slc38a1	Inpp5f	Sec11c	Dusp5	Klhl4
RP24-63O23.1	Zfp703	Tox	Npc2	Anks1	Cd164	Hivep1	Eprs	Tmeff1	Sc5d	Gab1	Idi1
Ctsh	Cit	Tmem164	Rnf144b	Lamb2	Gm17167	Lamp2	Tmem132a	Clic4	Hpca	Sqle	Litaf
Rgs6	Mef2c	Slc3a2	Cyp2d22	Plcxd2	Crip2	Pcdh10	Rgs4	Gm25939	Arhgef28	Ccp110	Apbb2
Abca1	Cckbr	Nrep	Rraga	Rps16	Myh9	Tmtc1	Camk2n1	Ankib1	Cabp7	Sgk1	Smco3
Slc13a4	H2-K1	Gucy1a3	Kcnb1	Tbc1d9	Gm26883	Nyap2	Pcsk1n	Arhgef25	Dhx33	Ldlr	42617
Irgm1	Gm5859	Ptgfrn	Arpp19	Anxa5	Rims4	Atp2b2	Camkk2	Olfml2b	Arrdc3	Drd5	Gm5067
Moxd1	Cd63	Mxd1	Aldh2	Cfh	Etv5	Hnrnpul2	Tubb4a	Cnih2	Gpr56	Msmo1	Abca8a

Supplemental Fig. 4 CNS cell-specific transcripts that were significantly altered by demyelination and partially restored by remyelination. Data for newly-formed oligodendrocytes, oligodendrocyte progenitors cells (OPCs), vascular endothelia, and non-specific cell types are shown in the heatmaps. Higher expression levels are indicated in red and lower expression levels are indicated in blue. The table below shows transcripts (192) expressed by multiple CNS cell types that were significantly altered by demyelination and partially restored by remyelination. Genes highlighted in red were increased by demyelination, while those highlighted in blue were decreased. Complete sequencing results are uploaded in the NCBI Gene Expression Omnibus (GEO) repository and can be downloaded with the appropriate accession number.

Supplemental Figure 5

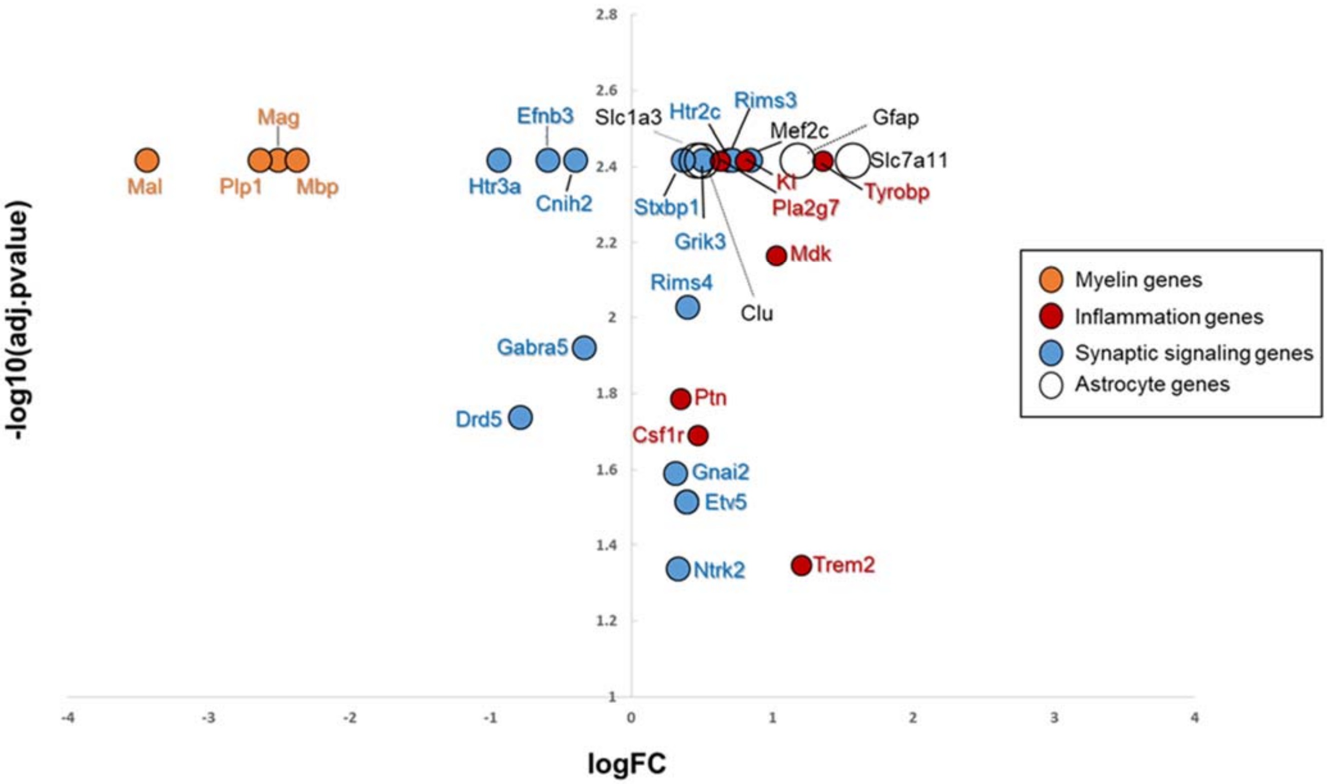

Supplemental Fig. 5 Volcano plot of selected gene transcripts that are significantly altered in demyelinated hippocampi. Myelin transcripts were decreased (orange circles), inflammatory (red circles) and astrocytic (white circles) transcripts were increased, while synaptic signaling transcripts (blue circles) were mixed.

Supplemental Videos

Supplemental Video 1 Serial EM sections used for dendritic spine reconstructions. Dendritic spines are colored yellow. Presynaptic terminals are colored blue.

Supplemental Video 2 Video showing reconstruction of spines projecting from a dendrite.
